# Incidental Dural Tears During Lumbar Spine Surgery: Prevalence and Evaluation of Management Outcomes

**DOI:** 10.7759/cureus.54212

**Published:** 2024-02-14

**Authors:** Monther Alessa, Faris Ababneh, Faisal Al Taimeh, Saad Haddad, Jeries Al Rabadi, Anees Hjazeen

**Affiliations:** 1 Department of Orthopedics, Royal Medical Services, Amman, JOR; 2 Department of Biostatistics, Royal Medical Services, Amman, JOR

**Keywords:** sandwich technique, prevalence, management, lumbar spine surgery, incidental dural tears

## Abstract

Introduction: Incidental dural tears (IDTs) are sometimes observed as an intraoperative complication associated with lumbar spine surgery. Commonly, this complication is recognized and repaired during surgery, but if it is undiagnosed or inadequately treated, a variety of consequences may occur. Many techniques have been developed to treat cerebrospinal fluid (CSF) leakage, and each has its limitations.

Objectives: To assess the prevalence of incidental dural tears in lumbar spine surgeries and evaluate the outcomes of the sandwich technique in the management of this complication.

Methods: A total of 92 patients who underwent lumbar spine surgery at the Royal Rehabilitation Center in Amman from January 2018 to December 2021 were retrospectively reviewed. Patients were divided into two groups: group A (patients without IDT) and group B (patients with IDT), where group B was repaired using the sandwich technique. The follow-up period was six months. Further, the sandwich technique involves repairing the dural defect with interlocking sutures, painting medical glue around the dural incision, covering this with gelatin sponge, and finally covering the gelatin sponge with medical glue again.

Results: The overall prevalence of IDT in the study group was 14.1%. IDT was more common among elderly patients above the age of 60 (17.2%), females (16.7%), patients with multiple lumbar levels treated (66.7%), open approaches (21%), and those who had previous spinal surgery (72.7%). Most IDTs were diagnosed and managed intraoperatively (84.6%). Among those patients, only one complained of a surgical site infection. Patients in group B had a significantly higher postoperative length of hospital stay, amount of drainage, and operative time compared to group A (P<0.001). Regarding postoperative pain, patients in group B had significantly higher pain on the Numerical Pain Scale at day three post-operation compared to patients in group A (P<0.001).

Conclusion: Based on our results, the sandwich technique was effective in the management and prevention of CSF leakage. Further prospective studies with long-term follow-up are needed to confirm our findings.

## Introduction

An incidental dural tear (IDT) is a relatively common complication during spinal surgery, with an incidence rate varying from 2.7% to 17.2% [1-3]. Many factors influencing the incidence of IDT have been reported in the literature, including surgical complexity [4], besides the surgeon's experience and patient factors, such as older age, sex, primary disease, and previous surgery [2]. Previous studies have shown that serious complications may occur as consequences of IDT, including surgical site infection, headache, nausea, vomiting, pseudomeningocele, external cerebrospinal fluid (CSF) fistula, meningitis, intracranial hemorrhage, and arachnoiditis with subsequent chronic pain [1,2]. Some of those complications are usually self-limiting within 10 days, such as headache and symptoms of meningism as a result of dural healing unless, development of a cerebrospinal fistula, which may result in a pseudomeningocele. Consequently, prolonged flat bed rest is required post-operation, which may lead to the development of pressure ulcers, deep venous thrombosis, and pulmonary embolism [5]. 

Thus, tears varying in size from pinpoint holes to several centimeters are often recognized and repaired during surgery; however, treatment for an IDT is contingent on the size of the tear as well as its accessibility. There have been several different methods of dural repair documented, from simple interrupted sutures, which are utilized to close simple durotomies, to cadaveric dura mater and fascia lata [6,7], and expanded polytetrafluoroethylene (ePTFE) sheet (Gore-Tex augmentation material). Each of those methods has been associated with such complexities, which range from prolonged surgery time-consuming during preparing the auto facia lata and suturing it to the dura or cost-effectiveness for the allograft facia lata and repairing dural tears with simple suturing alone, from our experience, will not provide sealed and secured dural defects. Simple interrupted sutures may fail to prevent cerebrospinal fluid (CSF) leakage, and it could be challenging in minimally invasive procedures; therefore, the development of nonpenetrating titanium clips solves this problem [8]. Because of the risk of Creutzfeldt-Jakob disease and other viral diseases among patients treated with cadaveric dura mater and fascia lata [6,7], allogenic human fascia lata was developed to avoid viral and bacterial transmissions in the early eighties.

Even though the effectiveness of expanded polytetrafluoroethylene in the management of dural tears was demonstrated, it was also associated with infection [9]. In the study reported here, we retrospectively reviewed the prevalence of IDT in lumber spine surgeries and evaluated the clinical effectiveness of the "sandwich" method in the management of this complication.

## Materials and methods

With the permission of the Ethics Review Committee of Jordanian Royal Medical Services, a retrospective cohort study design was used to review the files of patients' who underwent lumbar spine surgery at the Royal Rehabilitation Center from January 2018 to December 2021 for the following data: 1. Patients' characteristics, including age, sex, primary diseases, and previous spinal surgery; 2. Type of surgery, including lumbar spinal canal stenosis, degenerative lumbar spondylolisthesis, lumbar disk herniation, isthmic lumbar spondylolisthesis, lumbar degenerative scoliosis, posterior lumbar interbody fusion, transforaminal lumbar interbody fusion (TLIF), and others (posterolateral fusion, anterior spinal fusion); 3. Operation approach (open versus minimally invasive); 4. Occurrence of dural tears during surgery; 5. Post-operative parameters, such as postoperative length of hospital stay and post-operative complications, such as dural leak, prolonged bed rest, headache, nausea/vomiting, delayed wound healing, post-operative neurological deficit, surgical site infection, and reoperation for dural tears; 6. Clinical indicators include the Numeric Rating Scale (NRS), a pain rating scale that is used before and after surgery (third day). According to nursing policy at the Royal Rehabilitation Center, NRS is measured and written into the patient's medical file on a daily basis. 

Patients with intradural pathology requiring durotomy and patients with a traumatic dural tear (secondary to a spinal fracture) were excluded. Also, electronic medical files of patients who don't have the required information were left out.

Sampling technique

The non-probability consecutive sampling method was used to collect the historical patient data from those who met the specified study's inclusion and exclusion criteria within the study period, and then the patients were divided into two groups according to the occurrence of the IDT during surgery: group A (patients who had lumbar spine surgery without IDT occurrence) and group B (patients who had lumbar spine surgery with IDT occurrence).

Analysis of intraoperative (operation time) and post-operative parameters (amount of drainage, postoperative length of stay, and NRS post-operation) in patients with and without IDT was used to figure out the outcomes of the sandwich technique. Moreover, complications in patients with IDT were documented.

Surgical procedure

After the patient was in either the prone or supine position, general anesthesia was given. C-arm fluoroscopy was used to identify the precise locations of the lumbar spine. In the event of IDT occurring, the surgeon plugs the site with a cottonoid patty. Next, Vicryl sutures are used to repair the tear using interlocking sutures, and then we apply duraseal sealant. Followed by applying gel foam to cover the repair, and finally, duraseal sealant is used again over the foam. Duraseal is a type of synthetic absorbable sealant containing polyethylene glycol (PEG), which can improve the strength of sutured repair dura. Small tears (less than 1 cm) are treated with sutures, and then we apply duraseal sealant, gel foam, and duraseal sealant. For tears that are less than one centimeter in length (short tears), the sutures in the center section of the tear are cut, but the sutures at the end of the tear are left uncut, and the needles are left attached to the cut and removed after checking that the repair is sealed. When repairing longer tears, not only are the end sutures left uncut but also one or more of the intermediate sutures are left uncut. This makes sure that every centimeter of the repair has at least one uncut suture (we leave the sutures uncut for the possibility of adding facia lata after testing our repair by Valsalva maneuver, but fortunately we did not need to use any facia lata to our sandwich technique that has been used in repairing dural tears in group B).

Sample size estimation

The sample size was determined based on the Steven Thompson formula for observational studies. The population size for those who underwent lumbar spine surgery from January 2018 to December 2021 was 119 patients, with a z value for the 95% CI set at 1.96, a margin of error of 5.0%, and an assumed population proportion of 50.0%. The minimum required sample size was 92 patients

n = N × p(1−p)/ ((N−1 × (d2 × z2)) + p (1−p)), 

where N is the population size, z=1.96, p is the population proportion, d is the margin of error, and n is the sample size. 

Data analysis

The software IBM SPSS Statistics for Windows, Version 20 (IBM Corp., Armonk, New York) was utilized to perform the statistical analyses. Descriptive analysis is utilized to present sample characteristics. The operation parameter (operative time) and clinical outcomes (NRS post-op, post-op length of stay in hospital, and amount of drainage) between groups were compared using multivariate analysis (MANCOVA test) after controlling for the covariates (age and NRS pre-op). A P-value of 0.05 or less was established for statistical significance.

## Results

A total of 92 patients underwent various surgical procedures in the lumber spine during the study period and fulfilled the inclusion and exclusion criteria for the study. The overall prevalence of IDT was noted to be 14.1% (13 patients). When stratified by patient age, the prevalence of IDT varied from a minimum of 8.3% (one of 12 patients) among patients 30 to 39 years of age to a maximum of 17.2% (five of 29 patients) in those >60 years of age. The prevalence of IDT was higher in females (16.7%) compared to males (12%). The prevalence of IDT with regard to patients' demographic variables is summarized in Table 1. 

**Table 1:** The prevalence of IDT based on patients' demographic. n: number of cases, (%): percentage, IDT: incidental dural tears. Group A: Patients without incidental dural tears, Group B: Patients with incidental dural tears.

Demographic variables	Total no (%)	Group A, n=79 (85.9%)	Group B, n=13 (14.1%)
Age			
Less than 30 years	14 (15.2)	12 (85.7)	2 (14.3)
30 to 39 years	12 (13)	11 (91.7)	1 (8.3)
40 to 49 years	19 (20.7)	17 (89.5)	2 (10.5)
50 to 59 years	18 (19.6)	15 (83.3)	3 (16.7)
More than 60 years	29 (31.5)	24 (82.7)	5 (17.2)
Gender			
Male	50 (54.3)	44 (88)	6 (12)
Female	42 (45.7)	35 (83.3)	7 (16.7)

The majority of patients received a single-level of lumbar spine surgery (77.2%) and had no prior history of spinal surgery (88%). In 67.4% of cases, the operation was carried out utilizing an open approach. Incidental dural tears were commonly reported in patients with multiple lumbar levels (66.7%) and in those patients who had previous spinal surgery (72.7%). All IDT occurred among those patients who had an open-approach surgery. The prevalence of IDT with regard to operation data is summarized in Table 2.

**Table 2:** Prevalence of IDT based on operation data. n: number of cases, (%): percentage, IDT: incidental dural tears. Group A: Patients without incidental dural tears, Group B: Patients with incidental dural tears.

Operation variables	Total no (%)	Group A, n=79 (85.9%)	Group B, n=13 (14.1%)
Number of lumbar levels treated			
Single level	71 (77.2)	68 (95.8)	3 (4.2)
Double level	12 (13)	8 (66.7)	4 (33.3)
Multiple level	9 (9.8)	3 (33.3)	6 (66.7)
Surgery approach			
Minimal	30 (32.6)	30 (100)	0 (0)
Open	62 (67.4)	49 (79)	13 (21)
Previous spinal surgery			
No	81 (88)	76 (93.8)	5 (6.2)
Yes	11 (12)	3 (27.3)	8 (72.7)

The vertebral fracture was the most common indication among lumbar spinal surgeries (53.3%), followed by spinal canal stenosis, lumbar disc herniation, spondylolisthesis, extradural spinal tumor, and scoliosis in 16 (17.4%), 15 (16.3%), 8 (8.7%), 3 (3.3%), and 1 (1.1%) patients, respectively. Incidental dural tears were commonly reported in patients with lumbar disc herniation (33.3%) and extradural spinal tumor (33.3%) and least reported in patients with vertebral fracture (2%). The prevalence of IDT based on indications of lumbar spine surgery is summarized in Table 3.

**Table 3:** Prevalence of IDT based on indications of lumbar spine surgery. n: number of cases, (%): percentage, IDT: incidental dural tears. Group A: Patients without incidental dural tears, Group B: Patients with incidental dural tears.

Surgery indications	Total no (%)	Group A, n=79 (85.9%)	Group B, n=13 (14.1%)
Vertebral fracture	49 (53.3)	48 (98)	1 (2)
Lumbar disc herniation	15 (16.3)	10 (66.7)	5 (33.3)
Spinal canal stenosis	16 (17.4)	13 (81.3)	3 (18.7)
Spondylolisthesis	8 (8.7)	6 (75)	2 (25)
Extradural spinal tumor	3 (3.3)	2 (66.7)	1 (33.3)
Scoliosis	1 (1.1)	0 (0)	1 (100)

Diagnosis and management

Eleven out of 13 patients who had IDT were recognized intraoperatively, and all of them were managed and repaired immediately. After treating each patient in the same manner as was described earlier. Vancomycin wash was performed. Patients received subfascial drains, which were left in place for a total of three days. Postoperatively, those patients were required to remain on bed rest for 3.6 days (the range was two to eight days), depending on the size of the IDT. Prophylaxis with a broad-spectrum antibiotic (cefuroxime) is begun before surgery for a period of 24 hours, and it is maintained after surgery as part of the standard procedure followed at our spine department. In terms of post-operative complications, only one patient complained of a surgical site infection. 

Two patients with IDT were postoperatively diagnosed and received conservative therapy. These patients were required to remain on bed rest for 14 days and receive antibiotic therapy. One of those patients presented with wound infection, headache, nausea, vomiting, and low back pain, whereas the other one presented with headache, nausea, and low back pain. The time of diagnosis, management, and complications of IDT are summarized in Table 4.

**Table 4:** Time of diagnosis, management, and complications of IDT. CSF: cerebrospinal fluid, IDT: incidental dural tears.

Identification of dural tears	No. of cases	Management	Complications
Intraoperative CSF leak	11	Eleven patients were managed by immediate repair after recognition of CSF leak	One presented with wound infection
Postoperative CSF leak	2	Two patients managed conservatively	One presented with wound infection, headache, nausea, vomiting, and low back pain.
			One presented with headache, nausea, and low back pain

Operation outcomes

Groups A and B have the following adjusted mean with regard to operation time: 128.2 min and 195.5 min, respectively. Operation time was significantly lower in group A compared to group B, F (1, 88)=20.86, P<0.001, and 19.2% of the variation in operation time was explained by groups.

Groups A and B have the following adjusted mean with regard to post-op length of hospital stay: 5.908 days and 10.09 days, respectively. The postoperative length of hospital stay was significantly lower in group A compared to group B; F (1, 88)=37.04, P<0.001, and 29.6% of the variation in the postoperative length of hospital stay was explained by groups. On day three after surgery, the NRS-adjusted mean score was significantly lower in group A compared to group B (2.248 and 4.032, respectively). Furthermore, the difference was statistically significant (F (1, 88)=34.91, P<0.001), explaining 28.4% of the NRS variance. Regarding postoperative drainage, groups A and B have the following adjusted mean: 129.1 ml and 268.6 ml, respectively. The amount of drainage was significantly lower in group A compared to group B; F (1, 88)=15.11, P<0.001, and 14.7% of the variation was explained by groups. However, all group B patients were followed with MRI at six weeks with no CSF leakage or pseudomeningocele. Operation parameters and clinical outcomes are summarized in Table 5.

**Table 5:** Mean comparison of operation parameters and clinical outcomes between group A and group B. NRS: Numeric Rating Scale; partial (η2): partial eta-squared for effect size. Group A: Patients without incidental dural tears, Group B: Patients with incidental dural tears.

Variables	Groups		F-value	p-value	partial η2
	A	B			
Operation parameters					
Operation time (minutes)	128.2	195.5	20.86	<0.001	0.192
Clinical outcomes					
Post-operation length of hospital stay (days)	5.908	10.09	37.04	<0.001	0.296
NRS post-operation (third day)	2.248	4.032	34.91	<0.001	0.284
Amount of drainage (milliliters)	129.1	268.6	15.11	<0.001	0.147

## Discussion

CSF leakage following IDT is often regarded as the most serious complication that might arise during lumbar spine surgery. Due to the potential for negative postoperative consequences from even minor dural tears, prompt intraoperative diagnosis is typically crucial. Fortunately, it has been reported that when dural tears are detected during the surgical approach and effectively treated, there is no increase in morbidity or change in clinical outcome [10]. Variability is a challenge in the management of IDT. Location, size, tissue quality, and the presence of adhesions may all have an impact on the efficacy of management [11]. Moreover, evaluation of these parameters on an individual basis is essential to deciding on a postoperative bed rest period. To this day, there aren't any definitive treatment recommendations based on evidence-based guidelines.

Dural tears have been reported to occur at rates as high as 17.2% in the literature [1-3]. In our study, the rate (14.1%) is within the range described in the literature. The prevalence was variable across age groups, and it was much higher in older patients above the age of 60 years (17.2%). Previous studies support this finding [12,13]. Aging is a complex process associated with degenerative changes in the spine. Therefore, the probability of IDT increased as if the spinal dura was attached tightly to the surrounding structures, particularly to the yellow ligaments. In 119 surgical cases with symptomatic lumbar spinal stenosis, Yayama et al. examined histologically and immunohistochemically the degenerative changes and calcium crystal deposition in the ligamentum flavum of the lumbar spine. Evidence of ossification (deposited within the ligament) and a loss of elasticity were reported [14]. The presence of synovial cysts, as well as marked ossification of the yellow ligament and hypertrophied yellow ligament, was expected to be a major contributing factor for IDT in geriatrics undergoing multilevel laminectomy with non-instrumented fusions [4].

The incidence of IDT was higher in female patients (16.2%) compared to male patients (12%). Previous investigations have shown contradictory results about gender differences in complication rates. The effect of gender differences on the perioperative and postoperative rates of complications in patients who underwent single-level transforaminal lumbar interbody fusion (TLIF) was reported by Raheem et al., who documented that the rate of complications was more prevalent in females than in males, and the most reported complications were IDT [15]. However, Khechen et al. conducted a retrospective analysis among patients who had TILF, and they found no difference in the perioperative complication rate based on gender [16].

The influence of previous surgery on the increased rate of IDT is well studied [2,12,13]. The findings of our analysis were in concordance with the previous literature. This could be because of scar tissue from the previous surgery and the loss of anatomical landmarks. Our findings highlight the need for the surgeon to be aware of the increased risk of IDT during surgery for patients who have a previous history of spinal surgery and to exercise extra caution in areas of thick scar tissue.

In our analysis, the prevalence of IDT was greater in patients who received double or multiple levels of lumbar spine surgery compared to those who underwent a single level of surgery. Recent studies have identified the number of surgical levels as a risk factor for IDT during lumbar spine surgery [17]. This may be explained by the increased duration of surgery, longer time of dural sac exposure, and greater energy consumption of the surgeon, which may increase the risk for IDT.

Concerning surgical approaches, the literature comparing the various approaches to surgery with regard to IDT incidence is scarce [18]. The risk of IDT is often only mentioned in relation to open or minimally invasive surgery in the majority of studies. Notably, we only detected IDT in patients who had open surgery. In a prospective comparative study with 420 patients, Sharma et al. found a higher rate of IDT in patients who underwent an open approach compared to a minimally invasive approach (15.9% vs. 6.4%, respectively) [18]. In a minimally invasive approach, minimal tissue retraction is required; the surgeon can avoid midline scar tissue and fibrosis in previously operated patients; and a small amount of ligamentum flavum or lamina is removed to access the disc during discectomy, resulting in a lower chance of IDT.

In our study, 7.7% (two cases) of IDT were unrecognized during surgery and diagnosed postoperatively via magnetic resonance imaging. A similar finding was also reported by Tang et al [17]. A high incidence of unrecognized IDT was reported in the Koyama et al. study (26.7%) among patients with malignant spinal tumors due to the invasive and adhesive nature of the malignant tumors [19]. Due to the fact that the vast majority of patients were asymptomatic and delayed symptoms of CSF leakage, it is difficult to determine the exact prevalence of unrecognized IDT.

In our study, all recognized IDTs were managed via the sandwich technique. Small tears (less than 1 cm) are treated with suture, then we apply duraseal sealant (polyethylene glycol hydrogel), gel foam, and duraseal, while longer tears are treated with suture, then we apply duraseal, subcutaneous fascia or deep muscle fascia coverage, duraseal, and gel foam. The use of a graft over the site of a dural repair is not new; Eismont et al. advocated fascial grafts secured by interrupted sutures for larger dural tears and fat grafts for smaller tears [20]. The use of polyethylene glycol (PEG), a synthetic, absorbable hydrogel sealant, has the potential to accomplish watertight dural closure more effectively than conventional sutures or fibrin sealants [21]. However, it is known to expand postoperatively, causing stenosis and nerve root compression [22,23]. A recent systemic review evaluated the efficacy of different spinal dural strategies and revealed that the combination of primary suture, graft, and sealants resulted in the lowest rate of CSF leakage (5.5%) compared to primary suture and sealant (13.7%) and primary suture alone (17.6%) [24]. In our study, no CSF leakage was detected among patients who were diagnosed and managed during surgery. 

Postoperatively, the treatment included Trendelenburg position bed rest to reduce the hydrostatic pressure of the CSF, appropriate antibiotic selection (cefuroxime), regular dressing changes, monitoring of fluid and electrolyte balance, and follow-up MRI.

Operation time was compared between the groups, and patients with IDT had a higher operation time compared to patients without IDT. Desai et al. reported similar findings [25]. Furthermore, we assess changes in the outcomes of the Numerical Rating Scale (NRS) and length of stay between both groups postoperatively. Patients with and without IDT improved postoperatively with regard to NRS, with the greater improvement found in patients without IDT. The results indicate that the proper management of IDT has no negative effect on NRS. A similar finding was also reported in the Burgstaller et al. study [26]. Patients with IDT have a significant increase in postoperative length of hospital stay as compared to patients without IDT. A similar finding was also reported in previous studies [25,27].

Drainage is a contradictory issue in spinal surgery complicated with IDT since it’s utilized to avoid postoperative extradural hematoma [28], while other surgeons oppose it to prevent excessive CSF drainage, which is associated with the risk of neurological complications [29]. According to our protocol, drainage is recommended in the case of IDT. The amount of drainage was compared between the groups. Drainage was significantly higher in those with IDT compared to those without IDT.

Study limitations

As a retrospective study, it suffered from the inherent problems with collecting data, such as selection bias. This single-center study only involves lumbar spine surgeries; therefore, the sample size is small. There was limited follow-up and no evaluation of confounding variables such as diabetes or smoking. Regarding IDT, the location of IDT was not reported in the study.

## Conclusions

Incidental dural tears are a common complication in lumbar spine surgery. All patients diagnosed and managed intraoperatively in our retrospective analysis exhibited no symptoms of CSF leaking, and no patients complained of headaches or needed a second operation to revise the repair site, indicating that our approach was successful. Dural tears were associated with longer operative times, increased postoperative length of hospital stay, and increased amount of postoperative drainage. 
